# The evolution of adult pollen feeding did not alter postembryonic growth in *Heliconius* butterflies

**DOI:** 10.1002/ece3.8999

**Published:** 2022-06-27

**Authors:** Laura Hebberecht, Lina Melo‐Flórez, Fletcher J. Young, W. Owen McMillan, Stephen H. Montgomery

**Affiliations:** ^1^ 2152 Department of Zoology University of Cambridge Cambridge UK; ^2^ 1980 School of Biological Sciences University of Bristol Bristol UK; ^3^ 56292 Smithsonian Tropical Research Institute Gamboa Panama

**Keywords:** development, dietary protein, larval growth, life‐history evolution, nitrogen, nutritional trade‐off, resource allocation

## Abstract

For many animals, the availability and provision of dietary resources can vary markedly between juvenile and adult stages, often leading to a temporal separation of nutrient acquisition and use. Juvenile developmental programs are likely limited by the energetic demands of many adult tissues and processes with early developmental origins. Enhanced dietary quality in the adult stage may, therefore, alter selection on life history and growth patterns in juvenile stages. *Heliconius* are unique among butterflies in actively collecting and digesting pollen grains, which provide an adult source of essential amino acids. The origin of pollen feeding has therefore previously been hypothesized to lift constraints on larval growth rates, allowing *Heliconius* to spend less time as larvae when they are most vulnerable to predation. By measuring larval and pupal life‐history traits across three pollen‐feeding and three nonpollen‐feeding Heliconiini, we provide the first test of this hypothesis. Although we detect significant interspecific variation in larval and pupal development, we do not find any consistent shift associated with pollen feeding. We discuss how this result may fit with patterns of nitrogen allocation, the benefits of nitrogenous stores, and developmental limitations on growth. Our results provide a framework for studies aiming to link innovations in adult *Heliconius* to altered selection regimes and developmental programs in early life stages.

## Graphical abstract

*Heliconius* butterflies collect and digest pollen grains as adults—a novel source of amino acids which close relatives lack. The evolution of pollen‐feeding is hypothesized to have reduced the protein target for growing *Heliconius* larvae, enabling them to develop faster and thus spend less time in this vulnerable stage, and making the system a potential case study on shifting trade‐offs and constraints in different life stages. We formally test this hypothesis by comparing larval growth curves and life‐history traits across 6 Heliconiini species, and show that, while there is variation in developmental parameters, it is not explained by the presence of adult pollen‐feeding.

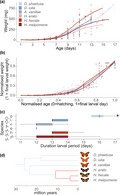

## 1 INTRODUCTION

Life‐history theory predicts that resource partitioning among life stages is optimized by both intrinsic life‐history trade‐offs and extrinsic environmental effects on survival and reproduction (Partridge et al., 2005; Reznick, 2010; Roff, 1992; Stearns, 1992). In holometabolous insects, ecological and biological differences between developmental periods can cause resource intake and utilization to become separated by life‐history transitions, making mechanisms of resource budgeting particularly important (Ahlström, 2011; Boggs, 1981; Istock, 1967). As the main foraging stage, larvae must therefore sustain immediate demands for growth and basal metabolism while building the reserves necessary for the pupal and adult stages (Boggs, 2009; Hahn, 2005).

In insects, the quantity and quality of the larval diet impacts a host of adult traits such as body size (Leftwich et al., 2017; Koyama & Mirth, 2018), lifespan (Min & Tatar, 2006; Grandison et al., 2009; Bruce et al., 2013; Runagall‐Mcnaull, Bonduriansky and Crean, 2015), survival (Sentinella et al., 2013; Rodrigues et al., 2015), reproductive output (Awmack & Leather, 2002; Hoover et al., 2006; Leather, 1995), and even behavior (Davies et al., 2018). However, larvae are also typically subject to high risks of predation, parasitism, and disease (Feeny, 1976; Gilbert, 1972, 1991). For example, several studies in tropical butterflies demonstrate high larval mortality (Smiley, 1985; Thurman et al., 2018), contrasting with low adult mortality seen in mark‐release‐recapture surveys (Turner, 1971; Cook et al., 1976; Gilbert, 1984; Mallet et al., 1987). Consequently, the duration of the larval period is subject to a trade‐off between resource acquisition and minimizing mortality (Dmitriew, 2011; Mattson, 1980; Nylin & Gotthard, 1998). This tension favors the evolution of optimal growth rates and “nutritional targets” (Raubenheimer & Simpson, 1993) which must be met before developmental transitions can be triggered. Larval nutritional targets are shaped by expected nutrient intake and expenditure during the adult phase (Boggs, 1981, 2009). Therefore, changes in adult diet are likely to alter patterns of resource requirements, unlocking potential for life history change (Houslay et al., 2015; Gray et al., 2018; Rostant et al., 2020). However, our understanding of the interplay between adult diet and life‐history evolution is limited to a small number of experimental evolution and comparative studies (O’Meara & Craig, 1970; Telang & Wells, 2004; Rostant et al., 2020).

Lepidoptera provide excellent opportunities to study how variation in resource partitioning can shape life‐history traits because they exhibit a variety of foraging ecologies as both larvae and adults (Slansky & Scriber, 1985; Swanson et al., 2016). As a resource, dietary nitrogen plays a crucial role in limiting developmental growth (Mattson, 1980) and constraining reproductive output (Fischer et al., 2004; Cahenzli & Erhardt, 2013; Swanson et al., 2016; Espeset et al., 2019). Since the majority of butterflies and moths feed on nectar (Slansky & Scriber, 1985), which is a poor source of amino acids (Baker & Baker, 1986), the nitrogenous requirements of adult butterflies and moths are largely provided for by nitrogen gathered during the larval phase from host‐plant tissue (O’Brien et al., 2002). One notable exception are *Heliconius* butterflies, which actively collect and digest pollen as a consistent source of amino acids during the adult stage (Gilbert, 1972; Young & Montgomery, 2020). The ability to pollen‐feed is a derived trait in *Heliconius*, marked by distinctive flower‐handling behaviors (Boggs et al., 1981; Penz & Krenn, 2000), foraging strategies (Gilbert, 1975), and mouthpart modifications (Krenn & Penz, 1998) absent in all other related genera. Pollen feeding in *Heliconius* brings about a substantial increase in expected adult nitrogen intake (Boggs, 1981), creating an evolutionary opportunity for change in patterns of juvenile resource allocation (Boggs, 2009). Gilbert (1972, 1991) and Boggs (1981) therefore predicted that the evolution of adult pollen feeding might have lifted constraints placed on the growth rates of *Heliconius* larvae by nitrogen nutritional targets, and that, relieved of these constraints, *Heliconius* may complete their larval development faster and thus minimize juvenile mortality. Indeed, previous studies in Heliconiini butterflies have highlighted differences in how pollen‐feeding *Heliconius* and nonpollen‐feeding outgroups allocate resources to reproduction during metamorphosis (Boggs, 1981; Dunlap‐Pianka et al., 1977), indicating that nitrogen targets in *Heliconius* larvae may indeed be reduced.

Here, we test one of the main predictions about the opportunity provided by the evolution of adult pollen feeding—namely, that *Heliconius* larvae will develop faster than closely related species with an exclusively nectar‐based adult diet. Specifically, we use a comparative approach to study the duration of larval and pupal development, larval growth curves, and pupal and adult weights of three pollen‐feeding *Heliconius* species and three nonpollen‐feeding Heliconiini.

## 2 METHODS

### 2.1 Animals

All individuals were reared between February and May of 2019 at the insectaries of the Smithsonian Tropical Research Institute in Gamboa, Panama, in ambient conditions. Outbred stock populations of six species were established: *Heliconius erato demophoon*, *H. melpomene rosina* and *H. hecale melicerta* (all pollen‐feeders), and *Dryas iulia*, *Agraulis vanillae*, and *Dryadula phaetusa* (exclusive nectarivores). Adults of each study species were collected within a 2 km radius of Gamboa and placed in 2 × 2 × 2 m cages to mate. All stock cages received fresh *Lantana* flowers daily, and artificial feeders with a 35% sugar solution. *Heliconius* stocks also received fresh *Psiguria* and *Gurania* flowers, and crushed bee pollen dissolved in the artificial feeders. Eggs were removed from host plants (Table S1) by hand each day, and stored without food until emergence. First, instar hatchlings were transferred to host plant shoots inside individually labeled cups and fed on their preferred host plants; *Passiflora biflora* (*H. erato*, *D. iulia*, and *D. phaetusa*), or *P. platyloba* (*H. hecale*, *H. melpomene* and *A. vanillae*), so as to favor optimal growth rates. In addition, to confirm our growth data were consistent with natural populations, body weight data were compared to data from wild‐caught adults collected in 2012 and 2013, measured as described in Montgomery et al. (2016).

### 2.2 Life history characterization

Larval hatchlings were weighed in groups of five, prior to feeding, with individual weight taken as one‐fifth of the combined weight. Larvae were then weighed individually once every two days to minimize handling‐related mortality. Individuals that successfully pupated were allowed to dry for 24 h and were then weighed. The duration of the larval and pupal periods was recorded in days. All individual weights were measured on a Sartorius H110 Handy Analytical Balance, with 0.1 mg resolution. Length of pupal period, but not weight, was also recorded for an additional 125 larvae, reared for other experiments.

### 2.3 Statistical analyses

All analyses were performed using R v.4.0.0 (R Core Team, 2020) and RStudio v.1.3 (RStudio Team, 2020). Unless stated otherwise, linear and generalized linear models (GLM) were generated with lme4 v.1.1‐23 (Bates et al., 2015). To test if larval development is shortened in pollen‐feeding species, we first assessed interspecific variation in the duration of the larval period (in days) by treating the data as counts and using GLMs with Quasi‐Poisson distributions to test the significance of species. *Heliconius* versus non‐*Heliconius* comparisons were then made to assess whether observed interspecific variation was primarily due to group differences, by means of mixed effect Conway‐Maxwell Poisson GLMs with species as a random factor, using the R package glmmTMB v.1.0.1 (Brooks et al., 2017). Additionally, linear models were built to test the robustness of the results, reported in Tables S2–S7. The same approach was followed to investigate variation in the duration of the pupal period.

We compared larval survival rates across species and adult foraging habits using the R package survival v. 3.2‐13 (Therneau & Grambsch, 2000). Larval body mass measurements were used to reconstruct growth curves and ascertain whether patterns of larval growth varied across species and groups. To better detect differences in growth patterns, we normalized developmental age across species for each individual to a range between 0 (hatching day) and 1 (day before pupation). Body mass was also normalized for each individual to a range between 0 and 1, with 1 equating to the last recorded larval weight. Since not all larvae were weighed on the last day before pupation, the difference in days between their final weight measurement and their final day as larvae was introduced as a random effect in the models. Linear models and GLMs with Gamma distribution and log‐link functions were built to compare growth curves, with individual and species as additional random effects. Models of normalized data with the lowest AIC scores are reported in the main text. Additionally, pairwise comparisons were run using the R package statmod v.1.4.34 (Giner & Smyth, 2016). Full results from normalized growth curve models, as well as equivalent models built on the original raw growth data, are presented in Tables S2–S7.

Finally, we detected interspecific variation in pupal and adult body weight using linear models, while linear mixed models with species as a random effect were used for *Heliconius* versus non‐*Heliconius* comparisons. Linear regressions with species as a random effect were then built to test whether the duration of the larval period correlated with pupal and adult weight.

## 3 RESULTS

### 3.1 Larval development

**FIGURE 1:**
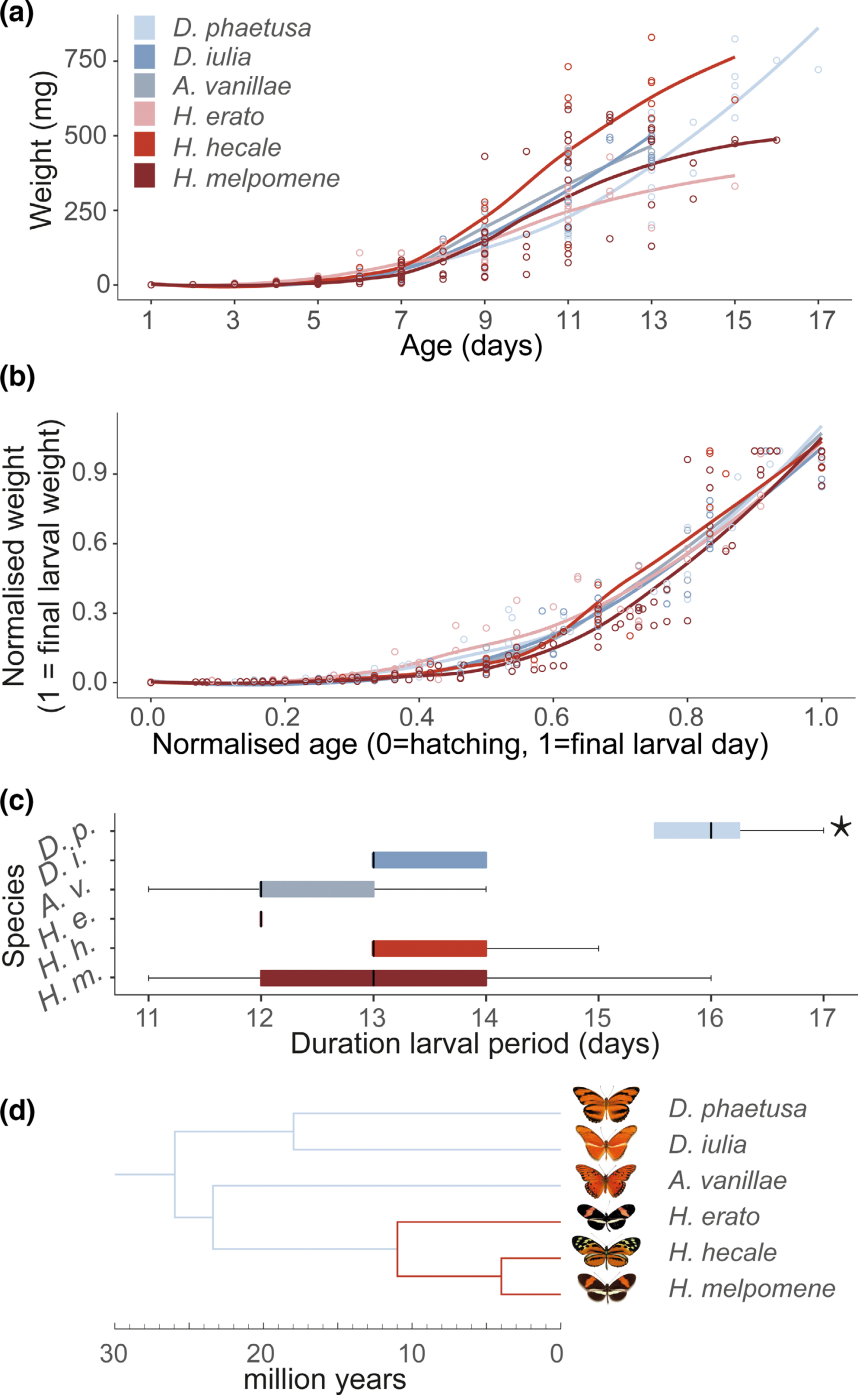
Larval growth curves and duration of larval development in pollen‐ (red tones) and nonpollen feeders (blue tones). (a) Variation in growth patterns and duration of larval period in raw larval weight data for 58 fully tracked individuals. Trend curves are generated with ggplot2 loess function and have no statistical purpose. (b) Variation in growth patterns and duration of larval period in normalized larval weight data for 58 fully tracked individuals as used in statistical comparisons. Growth patterns differ significantly between species (*F*_5,415_ = 3.202, *p* = .008) but not by adult foraging strategy (*F*_1,417_ = 0.292, *p* = .589). (c) Duration in days of the larval period per species. Duration of the larval period did not differ between pollen‐ and nonpollen feeders (*X^2^*_1,58_ = 0.398, *p* = .528). Asterisks denote significant interspecific contrasts at *p* < .0001 in posthoc comparisons. (d) Phylogenetic relationships between study species, adapted from Kozak et al. (2015)

We tracked the development of 125 larvae, of which 58 reached the 5th and final instar. Survival rates did not vary significantly across species (*X^2^*_5,125_ = 8.90, *p* = .100) or between pollen feeders and nonpollen feeders (*X^2^*_1,125_ = 1.00, *p* = .300). While we found significant variation in the duration of the larval period between species (*F*_5,52_ = 10.30, *p* < .0001), pollen feeders did not spend less time as larvae than nonpollen feeders (*X^2^*_1,58_ = 0.78, *p* = .379; Figure 1c; Table 1). Instead, interspecific variation in the duration of larval period was primarily driven by *D. phaetusa*, which had the longest developmental time (Table S2). When normalized to control for variation in the length of the larval period, larval growth curves (Figure 1a), which capture variation in the dynamics of the larval growth phase, also differ significantly between species (Figure 1b; *F*_5,57_ = 27.15, *p* < .0001). Although pairwise contrasts were inconsistent across models, growth curves of pollen‐feeding *Heliconius* consistently do not differ from their nonpollen‐feeding relatives (*F*_1,57_ = 0.05, *p* = .829; Table S3).

**TABLE 1:** Average duration of the larval development across species. Standard error = standard deviation/√*n*

Species	Pollen‐feeding	*N*	Mean duration (days)	Standard deviation	Standard error
*Dryadula phaetusa*	no	7	15.429	1.397	0.528
*Dryas iulia*	no	9	13.111	0.928	0.309
*Agraulis vanillae*	no	9	12.333	1.000	0.333
*Heliconius erato*	yes	10	12.100	1.101	0.348
*Heliconius hecale*	yes	9	13.444	0.726	0.242
*Heliconius melpomene*	yes	13	13.154	1.345	0.373

### 3.2 Pupal development

Fifty‐seven of the tracked larvae pupated successfully, and 46 completed pupal development. The weight of dried, fresh pupae varied across species (*F*_5,51_ = 17.74, *p* < .0001), but this variation was not explained by presence or absence of adult pollen feeding (*F*_1,3.98_ = 0.04, *p* = .845; Table S4). Heliconiini pupal development spanned 6 to 9 days, with *A. vanillae*, *H. erato*, and *H. melpomene* pupae developing significantly faster than *H. hecale*, and significantly slower than *D. iulia* (*F*_5,119_ = 17.51, *p* < .0001; Table 2; Table S5). Interspecific variation in the duration of the pupal period was not explained by pollen feeding (*X* ^2^_1,125_ = 0.38, *p* = .540).

**TABLE 2:** Average duration of the pupal period across species. Standard error = standard deviation/√*n*

Species	Pollen‐feeding	*N*	Mean duration (days)	Standard deviation	Standard error
*Dryadula phaetusa*	no	14	8.429	0.646	0.173
*Dryas iulia*	no	25	7.480	0.510	0.102
*Agraulis vanillae*	no	23	7.913	0.515	0.107
*Heliconius erato*	yes	29	7.690	0.541	0.101
*Heliconius hecale*	yes	12	9.000	0.000	0.000
*Heliconius melpomene*	yes	22	7.909	0.526	0.112

### 3.3 Adult weight and regressions

**FIGURE 2:**
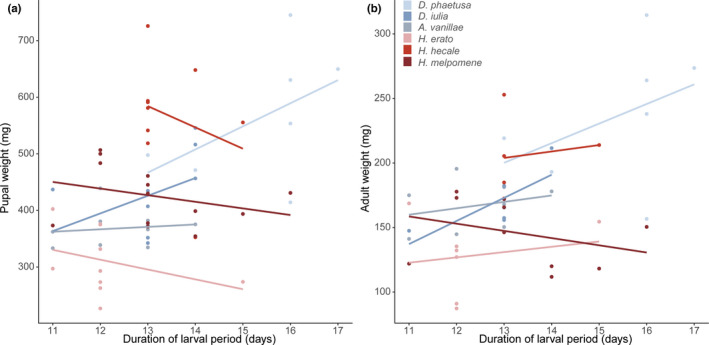
Correlations between pupal and adult weight and the duration of the larval period, by species. (a) There was no significant relationship between the length of the larval period and the final pupal weight when controlling for species (*F*_1,54.231_ = 0.470, *p* = .496). (b) There was no significant relationship between the duration of the larval phase and the final adult weight when controlling for species (*F*_1,40.48_ = 1.339, *p* = .254)

Forty‐three of the 46 surviving pupae emerged successfully. Adult *D. phaetusa* weighed significantly more than all other species except *H. hecale*, which were in turn significantly heavier than *H. erato* and *H. melpomene* (*F*_5,37_ = 11.06, *p* < .0001). As a group, adult body mass at emergence of *Heliconius* was not significantly different from that of the nectarivorous species (*F*_1,3.926_ = 0.97, *p* = .382; Table S6). Final larval weight was a significant predictor of both pupal and adult weight (larva/pupa *F*_1,19.796_ = 412.73, *p* < .0001, larva/adult *F*_1,41_ = 59.56, *p* < .0001), but there was no significant relationship between the duration of the larval period and body mass in future developmental stages (Figure 2; pupal body mass *F*_1,54.231_ = 0.47, *p* = .496, adult body mass *F*_1,40.688_ = 1.85, *p* = .181; Table S7). To confirm that our rearing conditions did not bias patterns of growth, we compared our adult weights to those of wild‐caught butterflies from the same population. We found that wild butterflies were lighter than caged ones (*F*_1,92_ = 20.65, *p* < .0001), but the ranks between species were conserved (*r*(4) = 1.00, *p* = .003).

## 4 DISCUSSION

By evolving to utilize a novel source of protein in the adult stage, *Heliconius* butterflies are hypothesized to have had an evolutionary opportunity to shorten the time spent as vulnerable larvae (Gilbert, 1972, 1991). Collecting pollen as adults, which provides a rich source of amino acids, could help recover the costs of accelerated larval growth and reduce the burden of foraging larvae to accumulate reserves for the adult stage. However, our results reject this effect in *Heliconius*. Developmental rates are subject to well‐documented trade‐offs with other life‐history traits (Nestel & Nemny‐Lavy, 2008; Cotter et al., 2011; Rádai et al., 2020). Since sampled individuals had access to unconstrained resources and preferred hostplants, our rearing treatment likely minimized plastic environmental effects governed by life‐history trade‐offs, and produced growth rates close to the maximum observed in the wild for each species. Under these conditions, we would expect to see evidence of any adaptive, developmental modifications related to the major adult dietary shift observed in *Heliconius*. While we observed significant species differences in larval and pupal developmental time, larval growth trajectories and pupal and adult weight, they were not associated with the presence of pollen‐feeding behavior in adults. This is not in line with patterns seen in other insects. For example, anautogenous strains of *Aedes spp*. mosquitoes, feeding on nutrient‐rich blood meals before ovipositing, spend less time in the larval phase than autogenous adult strains, which do not require a blood meal to oviposit, and therefore have greater reliance on larval stores (O’Meara & Craig, 1970). Our results also contrast with investment shifts already described in *Heliconius*. First, pollen‐feeding *H. charithonia* and *H. cydno* eclose with less total abdominal nitrogen, a proxy for larval reproductive investment, than the nonpollen‐feeding *Dryas iulia* (Boggs, 1981). Second, the ovaries of *H. charithonia* females are also smaller and contain fewer total oocytes than those of *D. iulia* females (Dunlap‐Pianka et al., 1977). These data strongly suggest that pollen feeders earmark relatively fewer larval reserves to reproduction, and if this energetic requirement were reallocated to the adult stage, this could favor the evolution of faster larval development in *Heliconius*. Below, we discuss three plausible alternative hypotheses that may explain why the energy diverted away from reproduction in *Heliconius* is not reallocated to larval growth rates.

First, *Heliconius* butterflies may use the larval reserves not invested in reproduction to strengthen their chemical defence (Cardoso & Gilbert, 2013). If so, the total nitrogen nutritional targets may remain the same for all Heliconiini larvae, and therefore limit growth rates across the board. Heliconiines are chemically defended by cyanogenic glycosides, both sequestered from their Passifloraceae host plants, and biosynthesized de novo from amino acids (Davis & Nahrstedt, 1987; Engler‐Chaouat & Gilbert, 2007). Comparisons across 19 Heliconiini show that *Heliconius* species are generally more cyanogenic than the other heliconiines (Pinheiro de Castro et al., 2019). However, this difference is nonsignificant, and nonpollen‐feeding *Agraulis vanillae*, as well as certain *Eueides* spp., show concentrations comparable to those detected in *Heliconius* species (Pinheiro de Castro et al., 2019). Such comparisons are complicated by the fact that concentration of cyanogenic glycosides alone might not be an accurate measure of toxicity level. Moreover, there is considerable interspecific variation in chemical profile (Pinheiro de Castro et al., 2019), with corresponding differences in the energetic costs of the cyanogenic metabolism. Further research is necessary to quantify energetic investment in toxicity throughout the life cycle, with particular focus on the allocation of larval nitrogen intake to cyanogenic metabolism.

Second, it may be that having large nutritional reserves upon emergence is advantageous for all Heliconiini, regardless of adult diet, particularly if the reliance on adult‐derived amino acids is “phased in.” Adult body size positively correlates with oviposition rates in *H. charithonia*, and is a direct consequence of larval food quality and quantity (Dunlap‐Pianka, 1979). Boggs and Iyengar, (2022) studied pollen use throughout the first 45 days of adult life in male and female* H. charithonia* in captivity. Male pollen load increased gradually until reaching a plateau around days 11–15, regardless of rearing environment. Females, on the other hand, delayed pollen collection until days 15–18 of adult life, unless they were fed ad libitum, in which case pollen‐collection trends resembled those of males. Trends in both sexes suggest that the role of pollen‐derived nutrition is less important during at least the first 10 days of adult life than it is thereafter, supporting a scenario in which it may be beneficial for young butterflies of either sex to emerge with large nutritional stores formed during the larval stage. Furthermore, oviposition rates of *H. charithonia* females reared without pollen only begin to drop after 15–20 days of pollen starvation (Dunlap‐Pianka et al., 1977) and are affected by the quality of larval diet (Dunlap‐Pianka, 1979). Similarly, for at least some *Heliconius* species, cyanogenesis is unaffected by pollen deprivation during the first 20 days after emergence, but declines thereafter (Cardoso & Gilbert, 2013), suggesting that cyanogenic metabolism also exploits larval reserves during early adult life. These data indicate that larval‐derived resources in *Heliconius* likely have a large role in supporting both reproduction and toxicity during the first weeks of adult life. This scenario appears to be at odds with cited evidence of comparatively less abdominal nitrogen and reproductive tissue in *Heliconius* than in nonpollen‐feeding *Dryas iulia* (Boggs, 1981; Dunlap‐Pianka et al., 1977). However, organism‐wide nitrogen contents in Boggs (1981, personal communication), are comparable between *H. cydno* and *D. iulia* (but not *H. charithonia*), suggesting that nitrogen not allocated to reproduction can be redirected elsewhere following adaptive pollen use, although this may not always be the case. More research is needed to track larval‐ and adult‐derived nitrogen in the products of reproductive and cyanogenic processes throughout the first month of the adult stage.

Third, even if *Heliconius* larvae do have a smaller nitrogen quota to fill, nitrogen accumulation may not be the limiting factor for faster larval development (Dmitriew, 2011). The larvae in this study were grown in their native environment under ambient conditions, with abundant supply of their preferred host plants and minimum incidence of disease. Therefore, we have likely observed the higher range of each species’ optimal growth rate. Our results may simply reflect the developmental constraints that cap all Heliconiini larvae under ideal growth conditions, and a faster development would not be possible without substantial costs (Arendt, 1997). For example, it may be that the developmental tasks shared by all Heliconiini, such as the accumulation of other nutrients, or the time and resources required to form the precursors of adult tissues or synthesize certain metabolites, are more time‐limiting than the formation of nitrogen reserves.

In conclusion, the simple prediction that increased quality of adult diets will relax constraints on *Heliconius* growth rates during vulnerable larval and pupal stages is not supported by our results. Indeed, current available evidence suggests that pollen feeding may have primarily relieved constraints on adult, rather than larval, development, allowing *Heliconius* to dramatically extend their lifespan (Ehrlich & Gilbert, 1973; Gilbert, 1972), sustain life‐long oogenesis (Dunlap‐Pianka et al., 1977), and potentially free nitrogenous resources for use in enhanced chemical defence (Cardoso & Gilbert, 2013; Young & Montgomery, 2020). To fully understand the consequences that the evolution of pollen feeding had on life‐history traits in *Heliconius*, it will be important to investigate trends in resource allocation and nitrogen use across the Heliconiini. In particular, it is unknown whether the larval nutritional target of pollen feeders is smaller than that of exclusive nectarivores, or if decreased investment in reproduction and increased investment in toxicity are characteristic traits in *Heliconius*. The fate of larval nitrogen throughout life stages has not been established, and the complicated landscape of heliconiine toxicity is only starting to be revealed. Finding answers to these questions will prime future use of the unique Heliconiini system as a tool to understand the coevolution of dietary innovations and life history components.

## AUTHOR CONTRIBUTIONS

**Laura Hebberecht:** Conceptualization (supporting); Data curation (equal); Formal analysis (lead); Investigation (supporting); Methodology (supporting); Supervision (supporting); Visualization (lead); Writing—original draft (lead); Writing—review & editing (equal). **Lina Melo‐Flórez:** Conceptualization (equal); Data curation (lead); Formal analysis (supporting); Investigation (lead); Methodology (lead); Visualization (supporting); Writing—original draft (supporting); Writing—review & editing (supporting). **Fletcher J. Young:** Conceptualization (equal); Data curation (equal); Formal analysis (supporting); Investigation (supporting); Methodology (supporting); Resources (supporting); Supervision (equal); Visualization (supporting); Writing—original draft (supporting); Writing—review & editing (supporting). **W. Owen McMillan:** Formal analysis (supporting); Project administration (equal); Resources (equal); Supervision (supporting); Visualization (supporting); Writing—original draft (supporting); Writing—review & editing (supporting). **Stephen H. Montgomery:** Conceptualization (equal); Data curation (supporting); Formal analysis (supporting); Investigation (supporting); Methodology (supporting); Resources (equal); Supervision (equal); Visualization (supporting); Writing—original draft (supporting); Writing—review & editing (equal).

## CONFLICT OF INTEREST

We declare no conflicts of interest.

## ETHICS

This work was carried out under permission from the Ministerio del Ambiente, Panama, Panama (permit numbers: SEX/A‐3‐12 and SE/AP‐14‐18).

## Supporting information

Table S1‐S7

Supplementary Material

## Data Availability

Data are available on the Data Dryad Digital Repository, https://doi.org/10.5061/dryad.3r2280gjn.

## References

[ece38999-bib-0001] Ahlström, T. (2011). Life‐history theory, past human populations and climatic perturbations. International Journal of Osteoarchaeology, 21(4), 407–419. 10.1002/oa.1147

[ece38999-bib-0002] Arendt, J. D. (1997). Adaptive intrinsic growth rates: An integration across taxa. The Quarterly Review of Biology, 72(2), 149–177. 10.1086/419764

[ece38999-bib-0003] Awmack, C. S. , & Leather, S. R. (2002). Host plant quality and fecundity in herbivorous insects. Annual Review of Entomology, 47(1), 817–844. 10.1146/annurev.ento.47.091201.145300 11729092

[ece38999-bib-0004] Baker, H. G. , & Baker, I. (1986). The occurrence and significance of amino acids in floral nectar. Plant Systematics and Evolution, 151(3–4), 175–186. 10.1007/BF02430273

[ece38999-bib-0005] Bates, D. , Mächler, M. , Bolker, B. , & Walker, S. (2015). Fitting linear mixed‐effects models using lme4. Journal of Statistical Software, 67(1), 1–48. 10.18637/jss.v067.i01

[ece38999-bib-0006] Boggs, C. L. (1981). Nutritional and life‐history determinants of resource allocation in holometabolous insects. The American Naturalist, 117(5), 692–709. 10.1086/283753

[ece38999-bib-0007] Boggs, C. L. (2009). Understanding insect life histories and senescence through a resource allocation lens. Functional Ecology, 23, 27–37. 10.1111/j.1365-2435.2008.01527.x

[ece38999-bib-0008] Boggs, C. L. , Smiley, J. T. , & Gilbert, L. E. (1981). Patterns of pollen exploitation by Heliconius butterflies. Oecologia, 48, 284–289. 10.1007/BF00347978 28309814

[ece38999-bib-0068] Boggs, C. L. , & Iyengar, V. (2022). Age‐specific and Sex‐specific Nectar and Pollen use by a Butterfly Pollinator. bioRxiv. 10.1101/2022.05.19.492749

[ece38999-bib-0009] Brooks, M. E. , Kristensen, K. , Benthem, K. J. , Magnusson, A. , Berg, C. W. , Nielsen, A. , Skaug, H. J. , Mächler, M. , & Bolker, B. M. (2017). glmmTMB balances speed and flexibility among packages for zero‐inflated generalized linear mixed modeling. The R Journal, 9(2), 378–400. 10.32614/rj-2017-066

[ece38999-bib-0010] Bruce, K. D. , Hoxha, S. , Carvalho, G. B. , Yamada, R. , Wang, H.‐D. , Karayan, P. , He, S. , Brummel, T. , Kapahi, P. , & Ja, W. W. (2013). High carbohydrate‐low protein consumption maximizes Drosophila lifespan. Experimental Gerontology, 48(10), 1129–1135. 10.1016/j.exger.2013.02.003 23403040 PMC3687007

[ece38999-bib-0011] Cahenzli, F. , & Erhardt, A. (2013). Nectar amino acids enhance reproduction in male butterflies. Oecologia, 171(1), 197–205. 10.1007/s00442-012-2395-8 22744741

[ece38999-bib-0012] Cardoso, M. Z. , & Gilbert, L. E. (2013). Pollen feeding, resource allocation and the evolution of chemical defence in passion vine butterflies. Journal of Evolutionary Biology, 26(6), 1254–1260. 10.1111/jeb.12119 23662837

[ece38999-bib-0013] Cook, L. M. , Thomason, E. W. , & Young, A. M. (1976). Population structure, dynamics and dispersal of the tropical butterfly Heliconius charitonius. The Journal of Animal Ecology, 45(3), 851. 10.2307/3584

[ece38999-bib-0014] Cotter, S. C. , Simpson, S. J. , Raubenheimer, D. , & Wilson, K. (2011). Macronutrient balance mediates trade‐offs between immune function and life history traits. Functional Ecology, 25(1), 186–198. 10.1111/j.1365-2435.2010.01766.x

[ece38999-bib-0015] Davies, L. R. , Schou, M. F. , Kristensen, T. N. , & Loeschcke, V. (2018). Linking developmental diet to adult foraging choice in Drosophila melanogaster. Journal of Experimental Biology, 221(9), 1–7. 10.1242/jeb.175554 29666197

[ece38999-bib-0016] Davis, R. H. , & Nahrstedt, A. (1987). Biosynthesis of cyanogenic glucosides in butterflies and moths. Insect Biochemistry, 17(5), 689–693. 10.1016/0020-1790(87)90037-0

[ece38999-bib-0017] Dmitriew, C. M. (2011). The evolution of growth trajectories: What limits growth rate? Biological Reviews, 86(1), 97–116. 10.1111/j.1469-185X.2010.00136.x 20394607

[ece38999-bib-0018] Dunlap‐Pianka, H. L. (1979). Ovarian dynamics in Heliconius butterflies: Correlations among daily oviposition rates, egg weights, and quantitative aspects of oögenesis. Journal of Insect Physiology, 25(9), 741–749. 10.1016/0022-1910(79)90126-4

[ece38999-bib-0019] Dunlap‐Pianka, H. , Boggs, C. L. , & Gilbert, L. E. (1977). Ovarian dynamics in heliconiine butterflies: Programmed senescence versus eternal youth. Science, 197(4302), 487–490. 10.1126/science.197.4302.487 17783249

[ece38999-bib-0020] Ehrlich, P. R. , & Gilbert, L. E. (1973). Population structure and dynamics of the tropical butterfly Heliconius ethilla. Biotropica, 5(2), 69. 10.2307/2989656

[ece38999-bib-0021] Engler‐Chaouat, H. S. , & Gilbert, L. E. (2007). De novo synthesis vs. sequestration: Negatively correlated metabolic traits and the evolution of host plant specialization in cyanogenic butterflies. Journal of Chemical Ecology, 33(1), 25–42. 10.1007/s10886-006-9207-8 17151910

[ece38999-bib-0022] Espeset, A. , Kobiela, M. E. , Sikkink, K. L. , Pan, T. , Roy, C. , & Snell‐Rood, E. C. (2019). Anthropogenic increases in nutrients alter sexual selection dynamics: A case study in butterflies. Behavioral Ecology, 30(3), 598–608. 10.1093/beheco/arz004

[ece38999-bib-0023] Feeny, P. (1976). Plant apparency and chemical defense. In J. W. Wallace & R. L. Mansell (Eds.), Biochemical interaction between plants and insects (pp. 1–40). Springer US. 10.1007/978-1-4684-2646-5_1

[ece38999-bib-0024] Fischer, K. , O’Brien, D. M. , & Boggs, C. L. (2004). Allocation of larval and adult resources to reproduction in a fruit‐feeding butterfly. Functional Ecology, 18(5), 656–663. 10.1111/j.0269-8463.2004.00892.x

[ece38999-bib-0025] Gilbert, L. E. (1972). Pollen feeding and reproductive biology of Heliconius butterflies. Proceedings of the National Academy of Sciences of the United States of America, 69(6), 1403–1407. 10.1073/PNAS.69.6.1403 16591992 PMC426712

[ece38999-bib-0026] Gilbert, L. E. (1975). Ecological consequences of a coevolved mutualism between butterflies and plants. In L. E. Gilbert & P. H. Raven (Eds.), Coevolution of animals and plants (pp. 210–240). University of Texas Press.

[ece38999-bib-0027] Gilbert, L. E. (1984). The biology of butterfly communities. In R. I. Vane‐Wright & P. R. Ackery (Eds.), The biology of butterflies. Symp Roy E. (pp. 41–54). Academic Press.

[ece38999-bib-0028] Gilbert, L. E. (1991). Biodiversity of a Central American Heliconius community: Pattern, process, and problems. In P. W. Price , T. M. Lewinsohn , G. W. Fernandes , & W. W. Benson (Eds.), Plant‐animal interactions: Evolutionary ecology in tropical and temperate regions (pp. 403–427). Wiley.

[ece38999-bib-0029] Giner, G. , & Smyth, G. K. (2016). Statmod: Probability calculations for the inverse Gaussian distribution. The R Journal, 8(1), 339–351. 10.32614/rj-2016-024

[ece38999-bib-0030] Grandison, R. C. , Piper, M. D. W. , & Partridge, L. (2009). Amino‐acid imbalance explains extension of lifespan by dietary restriction in Drosophila. Nature, 462(7276), 1061–1064. 10.1038/nature08619 19956092 PMC2798000

[ece38999-bib-0031] Gray, L. J. , Simpson, S. J. , & Polak, M. (2018). Fruit flies may face a nutrient‐dependent life‐history trade‐off between secondary sexual trait quality, survival and developmental rate. Journal of Insect Physiology, 104(May 2017), 60–70. 10.1016/j.jinsphys.2017.11.010 29203178

[ece38999-bib-0032] Hahn, D. A. (2005). Larval nutrition affects lipid storage and growth, but not protein or carbohydrate storage in newly eclosed adults of the grasshopper Schistocerca americana. Journal of Insect Physiology, 51(11), 1210–1219. 10.1016/j.jinsphys.2005.06.011 16098985

[ece38999-bib-0033] Hoover, S. E. R. , Higo, H. A. , & Winston, M. L. (2006). Worker honey bee ovary development: Seasonal variation and the influence of larval and adult nutrition. Journal of Comparative Physiology B: Biochemical, Systemic, and Environmental Physiology, 176(1), 55–63. 10.1007/s00360-005-0032-0 16228242

[ece38999-bib-0034] Houslay, T. M. , Hunt, J. , Tinsley, M. C. , & Bussière, L. F. (2015). Sex differences in the effects of juvenile and adult diet on age‐dependent reproductive effort. Journal of Evolutionary Biology, 28(5), 1067–1079. 10.1111/jeb.12630 25818561

[ece38999-bib-0036] Istock, C. A. (1967). The evolution of complex life cycle phenomena: An ecological perspective. Evolution, 21(3), 592. 10.2307/2406619 28563694

[ece38999-bib-0037] Koyama, T. , & Mirth, C. K. (2018). Unravelling the diversity of mechanisms through which nutrition regulates body size in insects. Current Opinion in Insect Science, 25, 1–8. 10.1016/j.cois.2017.11.002 29602355

[ece38999-bib-0038] Krenn, H. W. , & Penz, C. M. (1998). Mouthparts of Heliconius butterflies (Lepidoptera: Nymphalidae): A search for anatomical adaptations to pollen‐feeding behavior. International Journal of Insect Morphology and Embryology, 27(4), 301–309. 10.1016/S0020-7322(98)00022-1

[ece38999-bib-0071] Kozak, K. M. , Wahlberg, N. , Neild, A. F. E. , Dasmahapatra, K. K. , Mallet, J. , & Jiggins, C. D. (2015). Multilocus species trees show the recent adaptive radiation of the mimetic Heliconius butterflies. Systematic Biology, 64(3), 505–524. 10.1093/sysbio/syv007 25634098 PMC4395847

[ece38999-bib-0039] Leather, S. R. (1995). Factors affecting fecundity, fertility, oviposition and larviposition in insects. In S. R. Leather & J. Harvie (Eds.), Insect reproduction (pp. 143–174). CRC.

[ece38999-bib-0040] Leftwich, P. T. , Nash, W. J. , Friend, L. A. , & Chapman, T. (2017). Adaptation to divergent larval diets in the medfly, Ceratitis capitata. Evolution, 71(2), 289–303. 10.1111/evo.13113 27883361 PMC5324619

[ece38999-bib-0041] Mallet, J. , Longino, J. T. , Murawski, D. , Murawski, A. , & Gamboa, A. S. D. (1987). Handling effects in Heliconius: Where do all the butterflies go? The Journal of Animal Ecology, 56(2), 377. 10.2307/5054

[ece38999-bib-0042] Mattson, W. J. (1980). Herbivory in relation to plant nitrogen content. Annual Review of Ecology and Systematics, 11(1), 119–161. 10.1146/annurev.es.11.110180.001003

[ece38999-bib-0043] Min, K. J. , & Tatar, M. (2006). Restriction of amino acids extends lifespan in Drosophila melanogaster. Mechanisms of Ageing and Development, 127(7), 643–646. 10.1016/j.mad.2006.02.005 16616772

[ece38999-bib-0044] Nestel, D. , & Nemny‐Lavy, E. (2008). Nutrient balance in medfly, Ceratitis capitata, larval diets affects the ability of the developing insect to incorporate lipid and protein reserves. Entomologia Experimentalis Et Applicata, 126(1), 53–60. 10.1111/j.1570-7458.2007.00639.x

[ece38999-bib-0045] Nylin, S. , & Gotthard, K. (1998). Plasticity in life‐history traits. Annual Review of Entomology, 43(1), 63–83. 10.1146/annurev.ento.43.1.63 9444750

[ece38999-bib-0046] O’Brien, D. M. , Fogel, M. L. , & Boggs, C. L. (2002). Renewable and nonrenewable resources: Amino acid turnover and allocation to reproduction in Lepidoptera. Proceedings of the National Academy of Sciences of the United States of America, 99(7), 4413–4418. 10.1073/pnas.072346699 11930002 PMC123662

[ece38999-bib-0047] O’Meara, G. F. , & Craig, G. B. (1970). Geographical variation in Aedes atropalpus (Diptera: Culicidae). Annals of the Entomological Society of America, 63(5), 1392–1400. 10.1093/aesa/63.5.1392 5480641

[ece38999-bib-0048] Partridge, L. , Gems, D. , & Withers, D. J. (2005). Sex and death: What is the connection? Cell, 120(4), 461–472. 10.1016/j.cell.2005.01.026 15734679

[ece38999-bib-0049] Penz, C. M. , & Krenn, H. W. (2000). Behavioral adaptations to pollen‐feeding in Heliconius butterflies (nymphalidae, heliconiinae): An experiment using Latana flowers. Journal of Insect Behavior, 13(6), 865–880. 10.1023/A:1007814618149

[ece38999-bib-0050] Pinheiro de Castro, É. C. , Zagrobelny, M. , Zurano, J. P. , Zikan Cardoso, M. , Feyereisen, R. , & Bak, S. (2019). Sequestration and biosynthesis of cyanogenic glucosides in passion vine butterflies and consequences for the diversification of their host plants. Ecology and Evolution, 9(9), 5079–5093. 10.1002/ece3.5062 31110663 PMC6509390

[ece38999-bib-0051] Rádai, Z. , Kiss, J. , Babczyńska, A. , Kardos, G. , Báthori, F. , Samu, F. , & Barta, Z. (2020). Consequences of rapid development owing to cohort splitting: Just how costly is it to hurry? Journal of Experimental Biology, 223(6), jeb219659. 10.1242/jeb.219659 32098878

[ece38999-bib-0052] Raubenheimer, D. , & Simpson, S. J. (1993). The geometry of compensatory feeding in the locust. Animal Behaviour, 45(5), 953–964. 10.1006/anbe.1993.1114

[ece38999-bib-0053] Reznick, D. N. (2010). The ‘origin’ then and now. Princeton University Press. 10.2307/j.ctt7t064

[ece38999-bib-0069] R Core Team . (2020). R: A language and environment for statistical computing. R Foundation for Statistical Computing. https://www.R‐project.org/

[ece38999-bib-0054] Rodrigues, M. A. , Martins, N. E. , Balancé, L. F. , Broom, L. N. , Dias, A. J. S. , Fernandes, A. S. D. , Rodrigues, F. , Sucena, É. , & Mirth, C. K. (2015). Drosophila melanogaster larvae make nutritional choices that minimize developmental time. Journal of Insect Physiology, 81, 69–80. 10.1016/j.jinsphys.2015.07.002 26149766

[ece38999-bib-0055] Roff, D. (1992). Evolution of life histories. Theory and analysis (1st ed.). Chapman & Hall.

[ece38999-bib-0056] Rostant, W. G. , Mason, J. S. , de Coriolis, J.‐C. , & Chapman, T. (2020). Resource‐dependent evolution of female resistance responses to sexual conflict. Evolution Letters, 4(1), 54–64. 10.1002/evl3.153 32055411 PMC7006461

[ece38999-bib-0070] RStudio Team . (2020). RStudio: Integrated Development for R. RStudio, Inc. http://www.rstudio.com/

[ece38999-bib-0057] Runagall‐Mcnaull, A. , Bonduriansky, R. , & Crean, A. J. (2015). Dietary protein and lifespan across the metamorphic boundary: Protein‐restricted larvae develop into short‐lived adults. Scientific Reports, 5, 11783. 10.1038/srep11783 26119686 PMC4484247

[ece38999-bib-0058] Sentinella, A. T. , Crean, A. J. , & Bonduriansky, R. (2013). Dietary protein mediates a trade‐off between larval survival and the development of male secondary sexual traits. Functional Ecology, 27(5), 1134–1144. 10.1111/1365-2435.12104

[ece38999-bib-0059] Slansky, F. , & Scriber, J. M. (1985). Food consumption and utilization. In G. A. Kerkut & L. I. Gilbert (Eds.), Comprehensive insect physiology, biochemistry and pharmacology (Vol. 4, pp. 87–163). Pergamon Press.

[ece38999-bib-0060] Smiley, J. T. (1985). Heliconius caterpillar mortality during establishment on plants with and without attending ants. Ecology, 66(3), 845–849. 10.2307/1940546

[ece38999-bib-0061] Stearns, S. C. (1992). The evolution of life histories. Oxford University Press.

[ece38999-bib-0062] Swanson, E. M. , Espeset, A. , Mikati, I. , Bolduc, I. , Kulhanek, R. , White, W. A. , Kenzie, S. , & Snell‐Rood, E. C. (2016). Nutrition shapes life‐history evolution across species. Proceedings of the Royal Society B: Biological Sciences, 283(1834), 10.1098/rspb.2015.2764 PMC4947880 27412282

[ece38999-bib-0063] Telang, A. , & Wells, M. A. (2004). The effect of larval and adult nutrition on successful autogenous egg production by a mosquito. Journal of Insect Physiology, 50(7), 677–685. 10.1016/j.jinsphys.2004.05.001 15234628

[ece38999-bib-0064] Therneau, T. M. , & Grambsch, P. M. (2000). Modelling survival data: Extending the cox model. In Modelling survival data: Extending the cox model. Springer, US. 10.1007/978-1-4757-3294-8

[ece38999-bib-0065] Thurman, T. J. , Brodie, E. , Evans, E. , & McMillan, W. O. (2018). Facultative pupal mating in Heliconius erato: Implications for mate choice, female preference, and speciation. Ecology and Evolution, 8(3), 1882–1889. 10.1002/ece3.3624 29435261 PMC5792586

[ece38999-bib-0066] Turner, J. R. G. (1971). Experiments on the demography of tropical butterflies. II. Longevity and home‐range behaviour in Heliconius erato. Biotropica, 3(1), 21. 10.2307/2989703

[ece38999-bib-0067] Young, F. J. , & Montgomery, S. H. (2020). Pollen feeding in Heliconius butterflies: The singular evolution of an adaptive suite. Proceedings of the Royal Society B: Biological Sciences, 287(1938), 20201304. 10.1098/rspb.2020.1304 PMC7735275 33171092

